# A Prospective Study of Treatment Patterns and 1-Year Outcome of Asian Age-Related Macular Degeneration and Polypoidal Choroidal Vasculopathy

**DOI:** 10.1371/journal.pone.0101057

**Published:** 2014-06-30

**Authors:** Chui Ming Gemmy Cheung, Xiang Li, Ranjana Mathur, Shu Yen Lee, Choi Mun Chan, Ian Yeo, Boon Kwang Loh, Rachel Williams, Edmund Yick-Mun Wong, Doric Wong, Tien Yin Wong

**Affiliations:** 1 Vitreoretinal Service, Singapore National Eye Centre, Singapore, Singapore; 2 Singapore Eye Research Institute, Singapore, Singapore; 3 Department of Ophthalmology, Yong Loo Lin School of Medicine, National University of Singapore, Singapore, Singapore; 4 Ophthalmology Academic Clinical Program, Duke-NUS Graduate Medical School, Singapore, Singapore; 5 Department of Statistics and Applied Probability, National University of Singapore, Singapore, Singapore; 6 Worldwide Epidemiology, R&D Projects, Clinical Platforms & Sciences, GlaxoSmithKline, Pennsylvania, United States of America; Duke University, United States of America

## Abstract

**Objective:**

To study the treatment patterns and visual outcome over one year in Asian patients with choroidal neovascular membrane secondary to age-related macular degeneration (AMD-CNV) and polypoidal choroidal vasculopathy (PCV).

**Design:**

Prospective cohort, non-interventional study.

**Methods:**

132 treatment-naïve patients who received treatment for AMD-CNV and PCV were included. All patients underwent standardized examination procedures including retinal imaging at baseline and follow-up. AMD-CNV and PCV were defined on fundus fluorescein angiography and indocyanine green angiography at baseline. Patients were treated according to standard of care.We report the visual acuity (VA) and optical coherence tomography (OCT) measurements at baseline, month 3 and month 12 The factors influencing month 12 outcomes were analyzed.

**Main Outcome Measure:**

Type of treatment, number of Anti-vascular endothelial growth factor (VEGF) treatments, visual outcome over one year.

**Results:**

Anti-VEGF monotherapy was the initial treatment in 89.1% of AMD-CNV, but only 15.1% of PCV. The mean number of anti-VEGF injections up to month 12 was 3.97 (4.51 AMD-CNV, 3.43 PCV, p = 0.021). Baseline OCT, month 3 OCT and month 3 VA were significant in determining continuation of treatment after month 3. At month12, mean VA improved from 0.82 (∼20/132) at baseline to 0.68 (∼20/96) at month 12 (mean gain 6.5 ETDRS letters, p = 0.002). 34.2% of eyes (38/113 eyes) gained ≥15 ETDRS letters and 14.4% (16/113 eyes) lost ≥15 ETDRS letters. There were no significant differences in visual outcome between AMD-CNV and PCV (p = 0.51). Factors predictive of month 12 visual outcome were baseline VA, baseline OCT central macular thickness, month 3 VA and age.

**Conclusions:**

There is significant variation in treatment patterns in Asian eyes with exudative maculopathy. There is significant visual improvement in all treatment groups at one year. These data highlight the need for high quality clinical trial data to provide evidence-based management of Asian AMD.

## Introduction

Age-related macular degeneration (AMD) is one of the major causes of blindness worldwide [1]–[4]. The efficacy of ranibizumab, bevacizumab and aflibercept, have been confirmed by landmark clinical trials and these agents are now the mainstay of treatment for exudative AMD [5]–[11]. However, frequent follow-up and retreatment remain challenging in clinical setting. Data from the US Medicare and several European registries have highlighted these in the form of high treatment discontinuation rate within the first year and low mean number of injections [11]–[18].

There are few studies which have examined treatment pattern and outcomes of exudative maculopathy specifically in Asians, although it is often assumed that similar results with anti-VEGF therapy can be expected for eyes with choroidal neovascularization secondary to typical AMD (AMD-CNV) while different treatment appears to be required for the polypoidal choroidal vasculopathy (PCV) subtype [19]–[20]. In addition, lack of government funded reimbursement in many Asian countries, potential differences in patient understanding and expectation, and uncertainties of the role of anti-VEGF mono-therapy in PCV, may all affect the pattern of therapy in an Asian setting [20]–[24]. Furthermore, because photodynamic therapy (PDT) has been suggested to have superior angiographic, and possibly visual outcome in the PCV subtype [25]–[28], PDT is recommended as preferred treatment in PCV [29], in contrast to declining usage in Western populations. Therefore, significant heterogeneity remains in the management of Asian eyes with exudative maculopathy, in terms of diagnosis, optimal treatment and outcome.

There are few prospective studies which have examined treatment patterns and outcomes in a “real life” setting in Asians. To address this gap, we performed a prospective observational clinical study to document the current treatment pattern and outcomes of Asian exudative maculopathy, comparing in particular AMD-CNV and PCV.

## Methods

### Study Design and Population

The Asian AMD Phenotyping Study is a prospectively planned, cohort study approved by the SingHealth Centralized Institutional Review Board (protocol number R697/47/2009). All patients provided written informed consent to participate in this research. Specifically, the Asian AMD Phenotyping Study aimed to investigate prospectively a consecutive series of treatment-naïve Asian patients with exudative maculopathy secondary to AMD-CNV or PCV [30]. Consecutive patients were recruited from the retinal clinic of the Singapore National Eye Centre. Recruitment started on March 01 2010 and is still ongoing.

### Clinical Examination and Investigations

Participants received treatment according to standard of care by individual physicians, and treatment was not altered by entering into the study. Patients were followed up prospectively according to clinical need, but minimum reviews at month 1, month 3 and month 12 were mandated by the protocol to ensure minimal data collection. Additional visits were allowed if indicated by clinical needs. All patients underwent a complete standardized ophthalmic examination at baseline and follow-up. This included visual acuity, dilated fundus examination, color fundus photography and optical coherence tomography (OCT), fundus fluorescein angiography (FFA), and indocyanine angiography (ICGA), according to a standardized protocol at baseline.

Fundus photography was performed using a digital mydriatic retinal camera (TRC-50X/IMAGEnet 2000, Topcon, Tokyo, Japan). Spectral domain optical Coherence Tomography (OCT) was performed with the Cirrus OCT (Carl Zeiss Meditec, Dublin, CA) using the 512×128-volume cube setting. Central macular thickness (CMT) was recorded. Fundus angiography with fluorescein and indocyanine green is performed using a fundus camera (TRC-50X/IMAGEnet 2000, Topcon, Tokyo, Japan) or confocal SLO (Heidelberg Retina Angiograph Spectralis; Heidelberg Engineering, Heidelberg, Germany).

Best corrected visual acuity (BCVA) was tested with Snellen chart and converted to LogMAR. Repeat fundus photography and OCT were performed at each follow-up visit and repeat fluorescein and ICGA was performed as deemed appropriate by the treating retinal specialist.

### Angiographic Grading

AMD-CNV and PCV were diagnosed clinically and by FFA and ICGA. Both the primary diagnosis and treatment decision were made by the treating clinician, all of whom are fellowship-trained retinal specialist. Presence or absence of CNV was graded using criteria from the Macular Photocoagulation Study [31]. The diagnosis of definitive PCV lesions was based on the angiographic criteria from the Japanese Study Group guidelines, which defined PCV as characteristic polypoidal lesions on ICGA [32] To reflect the ‘real-world’ scenario, no secondary level grading was used in this analysis.

### Treatment Strategies

Treatment was recommended by one of the retinal specialists in the department, all of whom were fellowship-trained. However, patients decided their final treatment after considering their visual need and financial situation. Typically, anti-VEGF therapy was recommended in the presence of subretinal or intraretinal fluid or hemorrhage associated with active AMD-CNV. In cases of PCV, anti-VEGF was recommended where there was significant fluid or hemorrhage subfoveally. In addition, focal laser was recommended if polyps were localized extrafoveally. Where PCV lesions were extensive and not amenable to focal laser, PDT was recommended. Anti-VEGF monotherapy was used in patients who could not afford PDT for subfoveal or juxtafoveal PCV. In selected cases, individual retinal specialist might recommend anti-VEGF monotherapy if polyps were small and vision was good.

Patients were followed-up at intervals determined by their treating physician. A variety of regimens were used, including monthly with prn retreatment, treat-and –extend, and sometimes less frequent follow-up. Retreatment was based on visual acuity, clinical examination and OCT morphology, usually if there is persistent subretinal or intraretinal fluid or hemorrhage. Loading with 3 initial monthly injections was not compulsory.

Anti-VEGF treatment was given intravitreally either as ranibizumab (Lucentis, Novartis) 0.5 mg/0.05 ml or bevacizumab (Avastin, Roche) 1.25 mg/0.05 ml in pre-aliquoted syringes using local compounding pharmacy.

All focal laser treatment was performed by retinal specialists. Generally, treatment was performed with a green laser with a focal contact lens, using the following parameters: 100 to 200 µm spot size, 0.15 to 0.3 millisecond duration to achieve a greyish-white burn to the active polypoidal lesions identified on ICGA. Laser may be repeated at more than one session if closure is felt to be incomplete at follow-up. Laser was not performed on the associated branching network.

PDT was performed with verteporfin (Visudyne, Novartis, Basel, Switzerland) according to the protocol of the TAP study [33]. The greatest linear dimension (GLD) was measured based on FA for CNV lesions, according to TAP protocol. For eyes with PCV, the GLD included the entire area of abnormal choroidal vasculature on the ICGA. The diameter of the laser spot size selected to be 1000 µm more than the GLD. Patients were followed-up 3-monthly after the initial PDT.

### Statistical analysis

Statistical analysis was performed using standard statistical software (Statistical Package for Social Science, SPSS version 16, Illinois, Chicago) and R version 2.15.2 (R Development Core Team, Vienna, Austria, 2012). Characteristics of the study population were examined using proportions or means and standard deviation (SD). Wilcoxon rank sum test (Kruskal-Wallis rank sum test if number of groups is more than 2) or Chi-square test (Fisher's exact test if there is any entry with expected number less than 5) was performed to test the difference between diagnosis results (AMD-CNV vs. PCV) or between different treatment patterns. Factors influencing continuation of treatment after month 3 were analyzed by applying recursive partitioning tree model using R package “rpart” (Recursive Partitioning), to the factors of age, gender, initial treatment, OCT CMT (baseline, month 3 and change at month 3) and VA (baseline, month 3 and change at month 3). Decision tree was pruned after built to avoid overfitting.

Linear mixed-effect model was used to analyze longitudinal data of visual acuity over the first year to account for the repeated measurements made on the same eye. Both random and fixed effects of number of injections were added to control the heterogeneous effect among eyes. Stepwise model selection (forward and backward selection) was performed to investigate the effect on visual acuity at month 12, from a set of variables, including age, gender, VA at baseline, VA at month 3, change of VA at month 3, OCT CMT at baseline, OCT CMT at month 3, change of OCT CMT at month 3. Akaike information criterion (AIC) and recursive partitioning and regression tree model were used to select the important predictors for visual acuity at month 12.

## Results

From March 1^st^ 2010 to July 31^st^ 2011, a total of 167 study eyes from 167 patients were included (participation rate of 77.7%). The mean age of the subjects was 69.5 years, with a slight male dominance (58.1%). The presenting visual acuity was 0.85 (∼20/132). The proportion presenting with 0.3 (∼6/12) or better was 13.2%, and the proportion presenting with vision 1.0 (∼6/60) or worse was 35.3%. Sixty five eyes (38.9%) had typical AMD-CNV and 102 eyes (61.1%) had PCV. Baseline characteristics are summarized in **Table 1**.

**Table 1:** Baseline Characteristics, comparing eyes with Age-related Macular Degeneration (AMD-CNV) and Polypoidal Choroidal Vasculopathy (PCV).

	All (N = 167)	AMD-CNV (N = 65)	PCV (N = 102)	P
Age	69.5 (9.94)	70.47 (12.05)	68.88 (8.34)	0.08
Gender				
Men	97 (58.1%)	37 (56.9%)	60 (58.8%)	0.81
Ethnicity				
Chinese	145 (86.8%)	55 (84.6%)	90 (88.2%)	0.32
Indian	2 (1.2%)	1 (1.5%)	1 (1%)	
Malay	17 (10.2%)	9 (13.8%)	8 (7.8%)	
Others	3 (1.8%)	0 (0%)	3 (2.9%)	
Prior involvement of Fellow eye *	13 (7.8%)	8 (12.3%)	5 (4.9%)	0.08
Pseudophakia in affected eye	40 (24.1%) n = 166	20 (30.8%)	20 (19.8%) n = 101	0.11
**Visual Acuity (affected eye)**	0.85 (0.56),	**0.95 (0.55)**	**0.78 (0.57)**	**0.018**
% Visual Acuity 6/12 (0.3 LogMAR) or better	22 (13.2%)	6 (9.2%)	16 (15.7%)	0.23
**% Visual Acuity 6/60 (1.0 LogMAR) or worse**	59 (35.3%)	**29 (44.6%)**	**30 (29.4%)**	**0.045**
Visual acuity (fellow eye)	0.52 (0.64), n = 165	0.59 (0.7)	0.47 (0.59), n = 100	0.38
OCT CMT (affected eye)†	310.61 (98.51), n = 132	331.02 (106.33), n = 51	297.75 (91.61), n = 81	0.08
OCT CMT (fellow eye)	260.53 (80.05), n = 127	247.4 (65.14), n = 50	269.05 (87.75), n = 77	0.27

*Indicating whether a scar was present in the fellow eye.

† Exclude statistically outlier values of 18, 23, 785.

AMD-age-related macular degeneration, PCV- polypoidal choroidal vasculopathy, OCT- optical coherence tomography; CMT - central macular thickness.

### Initial Treatment Pattern for All Eyes

Treatment was initiated between baseline visit and 3-month visit in one hundred and thirty-two eyes (79%). At least 1 anti-VEGF injection was received by 97 of these 132 eyes (73.5%) during the first 3 months (**Table 2**). Of these 97 eyes treated with anti-VEGF, 54 eyes were as monotherapy, and the remainder was in combination with laser (12 eyes) or PDT (29 eyes) or both PDT and laser (2 eyes). After the initial 3 months, only 46.2% (61 eyes) received further treatment, most with continuation of anti-VEGF monotherapy (70.5%, 43 eyes). Data at 1 year was available from 87/97 eyes that were commenced on anti-VEGF therapy. The mean number of injections was 2.46 (3 months), 3.15 (6 months) and 3.97 (12 months) (**Table 3**). The mean cumulative numbers of out-patient visits were 3.08, and 8.03 at month 3 and month 12 (**Table 3**). Among eyes treated with any anti-VEGF, 83.2% received bevacizumab, and the remaining received ranibizumab. Thirty-four out of 41 patients initially treated with PDT remained on follow-up at one year. The mean cumulative number of PDT sessions was 1.29 (range 1–3) during the first year of treatment. Of the 35 eyes that initially did not receive treatment before their month-3 visit, 10 were eventually treated due to increase in activity or patient's change of mind, 8 patients defaulted and the remaining patients continued to be observed.

**Table 2:** Initial Treatment Pattern from Baseline to Month 3, comparing eyes with Age-related Macular Degeneration (AMD-CNV) and Polypoidal Choroidal Vasculopathy (PCV).

	All (N = 167)	AMD-CNV (N = 65)	PCV (N = 102)	P value†
**Initial treatment** *				0.036
No	35 (21%)	19 (29.2%)	16 (15.7%)	
Yes	132 (79%)	46 (70.8%)	86 (84.3%)	
				
**Eyes with initial treatment**	n = 132	n = 46	n = 86	<0.001
Any anti-VEGF	97 (73.5%)	44 (95.7%)	51 (59.3%)	
Anti-VEGF monotherapy	54 (40.9%)	41 (89.1%)	13 (15.1%)	
PDT monotherapy	9 (6.8%)	2 (4.3%)	7 (8.1%)	
Laser monotherapy	25 (18.9%)	0 (0%)	25 (29.1%)	
Anti-VEGF & PDT	29 (22.0%)	0 (0%)	29 (33.7%)	
Anti-VEGF & laser	12 (9.1%)	3 (6.5%)	9 (10.5%)	
PDT & laser	1 (0.8%)	0 (0%)	1 (1.2%)	
Anti-VEGF & PDT & laser	2 (1.5%)	0 (0%)	2 (2.3%)	

* Treatment given before month 3 (inclusive).

† P value to test the distribution of initial treatment between AMD and PCV, Fisher's exact test.

AMD-age-related macular degeneration, PCV- polypoidal choroidal vasculopathy, anti-VEGF- anti-vascular endothelial growth factor, PDT-photodynamic therapy.

**Table 3:** Cumulative number of Anti-vascular endothelial growth factor (anti-VEGF) injections and follow-up visits to month 12, comparing eyes with Age-related Macular Degeneration (AMD-CNV) and Polypoidal Choroidal Vasculopathy (PCV).

	Month 3	Month 6	Month 12
**All Anti-VEGF (N = 87)**			
**Number of injections**	2.46 (1.13)	3.15 (1.47)	3.97 (2.07)
**Number of visits**	3.08 (1.31)	4.11 (2.00)	8.03 (1.90)
**Stratified by Diagnosis & Treatment mode**			
**AMD-CNV (N = 43)**			
Number of injections	2.70 (1.19)	3.63 (1.63)	4.51 (2.25)
Number of visits	2.794 (1.41)	3.86 (2.17)	5.51 (2.76)
**AMD-CNV treated with Anti-VEGF monotherapy (n = 33)**			
Number of injections	2.73 (1.13)	3.55 (1.6)	4.45 (2.31)
Number of visits	2.52 (1.42)	3.33 (2.16)	4.91 (2.82)
**AMD-CNV treated with combination therapy (n = 10)**			
Number of injections	2.6 (1.43)	3.9 (1.79)	4.7 (2.21)
Number of visits	3.7 (0.95)	5.6 (0.97)	7.5 (1.27)
**P value** * **for number of injections comparing monotherapy vs combination in AMD-CNV**			
	1.000	0.536	0.511
**PCV (N = 44)**			
Number of injections	2.23 (1.03)	2.68 (1.14)	3.43 (1.73)
Number of visits	3.36 (1.16)	4.36 (1.81)	6.20 (2.36)
**PCV treated with Anti-VEGF monotherapy (n = 10)**			
Number of injections	3.1 (0.57)	3.8 (1.03)	4.8 (1.99)
Number of visits	3.7 (0.95)	5.2 (1.81)	7.3 (2.67)
**PCV treated with combination therapy (n = 34)**			
Number of injections	1.97 (1)	2.35 (0.95)	3.03 (1.45)
Number of visits	3.26 (1.21)	4.12 (1.75)	5.88 (2.2)
**P value** * **for number of injections comparing monotherapy vs combination in PCV**			
	0.002	0.001	0.008
**P value** * **for number of injections between AMD-CNV and PCV**			
	**0.003**	**0.002**	**0.017**

*P value based on Wilcoxon rank sum test.

AMD-age-related macular degeneration, PCV- polypoidal choroidal vasculopathy, anti-VEGF- anti-vascular endothelial growth factor, PDT-photodynamic therapy.

### One-Year Treatment Outcomes for All Eyes

**Figure 1:**
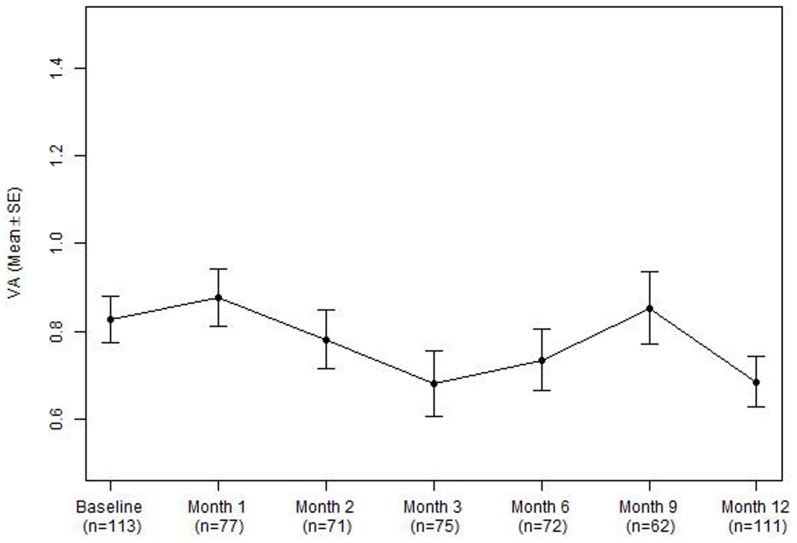
Mean visual acuity of all treated eyes from baseline to month 12. The number of eyes with OCT available at each timepoint is indicated by n.VA Visual acuity; SE standard error.

VA at month 12 was available in 113 (of 132) treated patients. The mean VA improved from 0.82 (∼20/132) at baseline to 0.68 (∼20/96) at month 12 (mean gain 6.5 ETDRS letters, p = 0.002, **Table 4** **,** **Fig 1**). 34.2% gained ≥15 ETDRS letters and 14.4% lost ≥15 ETDRS letters. VA at month 3 was available in 75 eyes (**Table 4**) and generally are similar to VA at month 12. At month 12, 48% of 98 eyes (including 58.5% of AMd-CNV eyes and 41.3% of PCV eyes) had activity when graded into categories of active and inactive. Central macular thickness measured using Cirrus OCT (available in 74 eyes) was significantly reduced from baseline to month 12 (300.86 µm vs 264.68 µm, p<0.001). The proportion of eyes with vision 0.3 (∼6/12) or better increased from 15.3% to 31.5%, and the proportion of eyes with vision 1.0 (∼6/60) or worse decreased from 33.3% at baseline to 22.5%. In the subgroup of 85 eyes treated with anti-VEGF, visual outcome was similar compared to the whole group, with mean gain of 7.0 letters at month 12. 32.9% (28/85) gained ≥15 letters and 12.9% (11/85) lost ≥15 letters.

**Table 4:** Visual Acuity and Optical Coherence Tomography Central Macular Thickness at Month 3 and Month 12, stratified by Diagnosis.

	All	AMD-CNV	PCV	P
**Visual acuity**	N = 113	N = 43	N = 70	
Baseline	0.82 (0.57)	0.79 (0.53)	0.83 (0.59)	0.89
Month 3	0.68 (0.65) (n = 75)	0.76 (0.79) (n = 25)	0.64 (0.57) (n = 50)	0.94
Month 12	0.68 (0.6)	0.7 (0.64)	0.67 (0.57)	0.94
Change at month 3	−0.16 (0.47)	0.03 (0.52)	−0.26 (0.42)	0.15
Change at month 12	−0.13 (0.51)	−0.09 (0.52)	−0.16 (0.51)	0.51
Gain ≥15 letters at Month 3	25 (33.3%)	9(36.0%)	16 (32.0%)	0.73
Gain ≥15 letters at Month 12	38 (34.2%)	14 (34.1%)	24 (34.3%)	0.99
Loss ≥15 letters at month 3	7 (9.3%)	6 (24.0%)	1 (2.0%)	0.005
Loss ≥15 letters at Month 12	16 (14.4%)	6 (14.6%)	10 (14.3%)	0.96
**Proportion with VA 0.3 or better**				
Baseline	17 (15.3%)	7 (17.1%)	10 (14.3%)	0.69
Month 3	29 (38.7%	11 (44.0%)	18 (36.0%)	0.50
Month 12	35 (31.5%)	14 (34.1%)	21 (30%)	0.65
**Proportion with VA 1.0 or worse**				
Baseline	37 (33.3%)	13 (31.7%)	24 (34.3%)	0.781
Month 3	17 (22.7%)	6 (24.0%)	11 (22.0%)	0.85
Month 12	25 (22.5%)	11 (26.8%)	14 (20%)	0.406
**OCT CMT**	N = 76	N = 26	N = 50	
Baseline	300.86 (98.29)	317.62 (94.79)	292.14 (99.88)	0.26
Month 12	264.68 (89.82)	272.81 (71.18)	260.46 (98.54)	0.16
Change at month 12	−36.17 (93.99)	−44.81 (79.52)	−31.68 (101.17)	0.68

P value based Wilcoxon rank sum test or Chi-square test.

AMD-age-related macular degeneration, PCV- polypoidal choroidal vasculopathy, OCT- optical coherence tomography, CMT - central macular thickness.

### Comparison of Treatment Patterns for AMD-CNV and PCV

Presenting vision was significantly worse in AMD-CNV than PCV (0.95 vs 0.78, p = 0.018) (**Table 1**) and more eyes with AMD-CNV had vision worse than 6/60 (44.6%) than PCV (29.4%, p = 0.045). However, within the group of treated eyes with at least one year follow-up (n = 113), the baseline vision was similar. There was no significant difference in age, gender, OCT thickness, proportion of previous involvement in fellow eyes and lens status. However after excluding 35 patients who did not receive treatment (29 AMD-CNV, 16 PCV), the presenting vision was similar in the remaining eyes (0.79 AMD-CNV, 0.83 PCV, p = 0.89) (**Table 4**).

**Figure 2:**
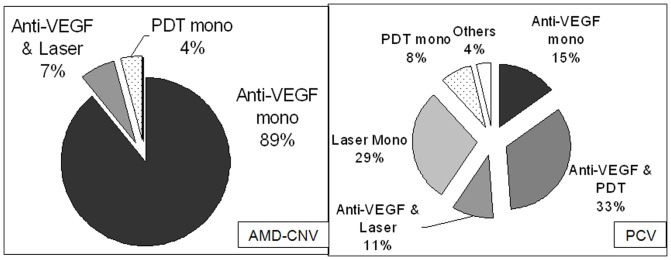
Distribution of Initial Treatment Modalities by diagnosis of Age-related Macular Degeneration (AMD-CNV) (left) and Polypoidal Choroidal Vasculopathy (PCV) (right).

Initial treatment choice was markedly different between AMD-CNV and PCV groups (**Table 2**, **Figure 2**). Anti-VEGF monotherapy was used in the majority of AMD-CNV (89.1%, 41/46 eyes), but only 15.1% (13/86) of PCV. Within the PCV group, the single most commonly used treatment was anti-VEGF and PDT combination (33.7%, 29/86), followed by laser monotherapy (29.1%, 25/86). Overall, 59.3% of eyes with PCV received anti-VEGF therapy alone or in combination. The mean cumulative number of anti-VEGF injections was significantly lower in PCV group (4.51 for AMD-CNV, 3.43 for PCV, p = 0.021).

Mean vision at month 12 was similar between AMD-CNV and PCV group (0.70 AMD-CNV, 0.67 PCV, p = 0.94) (**Table 3**). The mean change in vision and proportion gaining and losing ≥15 ETDRS letters were also similar. (**Table 3**)

### Factors Influencing Treatment Patterns

Factors that influenced the choice of initial therapy were diagnosis (see above, AMD-CNV compared to PCV) and OCT CMT at baseline. Eyes treated with anti-VEGF monotherapy had thicker OCT CMT at baseline compared to eyes treated with combination. (367.62 µm vs 291.68 µm, p = 0.001). These two factors remained significant (diagnosis, p<0.001; OCT CMT p = 0.004) after adjusting for age, gender, baseline OCT and diagnosis Baseline VA and previous fellow eye involvement were not significantly related to treatment choice.

Factors that influenced the cumulative number of injections included diagnosis, choice of initial treatment and OCT CMT measurement at month 3. PCV eyes had significantly less injections than AMD eyes (3.43 vs 4.51 at 1 year, p = 0.021) This difference remained after controlling age, gender and the OCT CMT at month 3. Eyes treated with anti-VEGF combinations had significantly lower number of injections than eyes treated with anti-VEGF monotherapy only in the PCV group (3.03 vs 4.70 at 1 year, p = 0.008). Each 100 µm increase in OCT CMT at month 3 was associated with additional 0.925 injections after month 3 (p = 0.029).This remains significant after controlling for diagnosis and the baseline OCT CMT (p = 0.025).

### Factors Influencing One-Year Visual Outcome

Using recursive partitioning and regression tree method, baseline VA, baseline OCT thickness and month 3 VA were significant predictors of VA at month 12. Eyes with baseline VA of ≥1.0 had worse vision at month 12 compared to eyes with better baseline VA (1.13 vs 0.46, p<0.001). However they experienced larger improvement in VA (18.5 letters vs 1 letter, p = 0.002) and had a larger proportion experiencing at least 15 letters gain (54.1% vs 24.3%, p = 0.002).

In addition, we performed a longitudinal analysis, utilizing all available data at different time points over the first year. Visual acuity was weakly correlated with number of injections and age. For each additional injection received, there was on average an additional 1.7 letters improvement in VA at month 12 (p = 0.05). Every year increase in age was associated with a 0.6 letter worsening of VA (p = 0.05). OCT CMT measurement was significantly correlated with visual acuity (p<0.001). Each 100 µm increase in OCT CMT was correlated to a drop of 3.62 ETDRS letters in visual acuity. There was no significant difference in Month 12 VA or change in VA whether anti-VEGF therapy was alone or in combination.

## Discussion

This prospective study reports the current treatment pattern and outcome for exudative maculopathy in an Asian population over a one year period. While it has been documented that anti-VEGF monotherapy is now the most common treatment for exudative AMD in the USA and Europe [11], similar data from an Asian population are limited. In addition, it is unclear how anti-VEGF usage may be affected by issues including the high prevalence of PCV in Asian populations, the apparent superior efficacy of PDT in causing polyp regression, and the uncertainty of the role of anti-VEGF monotherapy [20]–[29]. Recently, health authorities from some Asian countries have excluded PCV from the treatment label of anti-VEGF agents. Therefore, a clearer understanding of the management patterns and outcome of Asian eyes with exudative maculopathy is important in accurately planning eye care provision, research strategies, labelling of medications and re-imbursement of therapies.

Our study population can be compared to similar registry and insurance-based data, mostly from Western countries with largely white populations (**Table 5**). Our study population was generally younger (mean age 69.6 years), had higher male subjects (58.1%) and had lower presenting vision (44.0 ETDRS letters). Overall, 72.0% of treated eyes received at least one anti-VEGF treatment. While the widespread use of anti-VEGF is comparable to data from US [11], only 40.9% of cases received anti-VEGF as monotherapy. This pattern is remarkably different compared to reports in white people [11]. However, when stratified by diagnosis, the vast majority of AMD-CNV cases were treated with anti-VEGF monotherapy (89.1%). Therefore the lower monotherapy rate can be attributed largely to the higher proportion of patients needing specific PCV treatment, of which only 15.1% received anti-VEGF monotherapy. Despite the lower proportion of anti-VEGF monotherapy, a further 44.2% of PCV eyes received anti-VEGF in combination with PDT (33.7%) or laser (10.5%). Thus, an key observation of this study is that anti-VEGF therapy constitutes a significant part of treatment for PCV eyes (59.3%), although more commonly in combination with other treatments.

**Table 5:** Comparison of Patient Characteristics and Anti-VEGF Utilization during the First Year of Treatment.

Study origin	USA (2008) [11]	Germany (2008) [12], [13]	UK (2007) [14]	UK (2007) [14]	Sweden (2007) [12], [15]	Belgium (2008) [12], [16]	Netherlands (2008) [12], [16]	France (2007) [17]	Beirut (2005) [18]	Korea (2007) [19]	Singapore, Current study (2010)	Singapore, Current study (2010)
Sample size	91,628	3,470	897 (pretreated n = 125)	897 (no pretreatment n = 772)	471	253	243	122	60	41	132 (AMD-CNV, n = 43)	132 (PCV, n = 87)
Mean age, years (SD)	81.1 (7.0)	77.6 (7.8)	75.0 (8.4)		78.1 (8.0)	78.7 (6.8)	77.9 (8.0)	78.3 (7.0)	72.2	70.3 (7.9)	69.6 (10.5)	
Female, %	63.8%	64.6%	-		66.0%	62.1%	59.3%	70.0%	43.1%	51.2%	41.9%	
Mean baseline VA ETDRS letters (SD)	NA	48.8 (18.7)	50.4	54.1	58.3 (12.2)	56.3 (14.2)	45.1 (21.5)	56.2 (14.0)	45.7	42.1	45.5	43.5
Mean VA at 1 year ETDRS letters (SD)	NA	48.0 (11.7)	53.1	57.9	59.3 (16.2)	58.8 (17.9)	50.7 (24.0)	56.9 (17.0)	53.1	46.0	50.0	51.5
Mean number of injections at 1 year (SD)			6.2 (2.6)	5.2 (2.7)							4.51 (2.25)	3.43 (1.73)

Formula of conversion from LogMAR to ETDRS letters: 85-50LogMAR.

AMD - age-related macular degeneration; PCV- polypoidal choroidal vasculopathy; SD standard deviation.

The overall number of injections was low at 3.97 (range 1–9) over 12 months. Even after separating PCV eyes from AMD-CNV eyes, the mean number of injections was only 4.51 injections over 12 months in AMD-CNV eyes. However, these figures are not far off published data from USA and European registries (**Table 5**) [11]–[18]. These data highlight the challenges in translating clinical trial results into “real life” clinical practice. As in most countries, the utilization of anti-VEGF is affected by reimbursement structure and retreatment criteria. In addition to low retreatment number, the number of follow-up visits during the first year was also low (mean 8 visits). Although only three mandatory visits were included in the study protocol (at months 1, 3 and 12), most patients attended much more frequently due to clinical need (mean 8.03 visits). However,it is likely this lack of follow-up and under-treatment contributed significantly towards the low injection numbers, as 48% of eyes still had activity on OCT at month 12, However, similar low follow-up visits (8.06 visits) had been reported from France [17], highlighting the adherence to follow-up is also a challenge in the delivery of AMD care.

We report the mean VA improved significantly by 6.5 letters, with 34.2% gaining 15 ETDRS letters and 14.4% losing ≥15 ETDRS letters. When outcome was analyzed by diagnosis of AMD-CNV versus PCV, there was no significant difference in mean VA change, or of the proportion gaining and losing 15 letters. Visual outcome from several European registries have been summarized in **Table 5** for comparison. In addition to mean change in VA, it is also important to note that patients from the current study had generally worse presenting visual acuity than those from European registries [12]–[18]. Despite significant mean visual gain, the vision at 1 year remained significantly lower than those from European registries [12]–[18]. Lower public awareness of AMD and generally later presentation in Asian setting is likely to be a significant explanation for these findings. It is also interesting to note that 35 patients did not receive treatment initially (before their month-3 visit). The most common reasons were poor prognosis (10/19 eyes in the AMD-CNV group and 6/15 eyes in the PCV group), and limited activity at presentation (3/19 in the AMD-CNV group and 8/15 in the PCV group) reason in the PCV group (8/15 eyes). The remaining patients defaulted their follow up. Of these 35 eyes, 10 eyes eventually received treatment from month 4 onwards.

In terms of predictive factors for visual outcome, we identified OCT measurement at baseline and VA at month 3 as significant predictors for both the need to continue treatment beyond month 3 and also vision at month 12. Each 100 µm increase in OCT CMT was correlated to a drop of 3.6 ETDRS letters in visual acuity (p<0.001). OCT CMT measurement at month 3 was also predictive of whether treatment was continued after 3 months. Each 100 µm increase in OCT CMT at month 3 was associated with 0.925 additional injections after month 3 (p = 0.025). The number of injection was only weakly correlated with visual outcome at month 12. Each additional injection was associated with a further 1.7 letters gain in vision at month 12.

In our study, 61.1% of eyes had PCV. Apart from features on ICGA and worse baseline VA in AMD-CNV (0.95 in AMD-CNV vs 0.78 in PCV, p = 0.018), there were no significant differences in baseline characteristics in terms of age, gender, fellow eye involvement or lens status between AMD-CNV and PCV eyes. These results highlight the importance to screen for PCV with ICGA in an Asian population. Our results showed several major findings regarding PCV treatment in our centre. First, a variety of treatment modalities are currently employed in treating PCV. Second, anti-VEGF use was common, but appears to require less injection number when used as combination therapy. Third, the visual outcome was similar in PCV and AMD-CNV eyes in the anti-VEGF era. Therefore, the role of anti-VEGF therapy in PCV, as monotherapy or as adjunct to occlusive therapies, should be further studied.

There are limitations to this study. The observational design aims to capture the current treatment pattern of exudative AMD in an Asian setting in Singapore. Variations in treatment preference between individual clinicians, between countries and regions are to be expected. Variations in reimbursement systems between countries will also influence the treatment pattern. Practice pattern, such as injection number, is likely to evolve with time and injection numbers may increase with increasing acceptance of the need for long-term therapy. We did not perform secondary grading of PCV but decided to follow the initial clinical diagnosis by the treating clinician. Therefore PCV diagnosis may be less stringent, and some of the PCV cases in this series may represent possible/probable PCV (mixed cases) in addition to definite PCV cases. Due to logistic constraints, OCT examination was performed using SD-OCT other than the Cirrus OCT in some cases. Therefore the OCT thickness measurements in these eyes were not incorporated into the current analysis. Post treatment ICGA would have been informative in all cases. However ICGA was only repeated post treatment if there is clinical suspicion (based on fundoscopy, visual aculty and OCT findings) of residual activity.

In conclusion, our prospective study showed significant variation in treatment patterns in Asian eyes with exudative maculopathy. AMD-CNV was treated predominantly with anti-VEGF monotherapy, whereas a variety of treatment modalities were used to treat PCV. Low overall injection numbers over 1 year suggest that many patients may be under-treated in the “real life” setting, a situation not dissimilar to US and European countries. Despite these variations, relatively good visual outcomes at 1 year were achieved in both AMD-CNV and PCV. Nevertheless, these data highlight the need for high quality clinical trial data to provide evidence-based guidelines to optimize the management of Asian AMD.
